# Investigation of *KIF6* Trp719Arg in a Case-Control Study of Myocardial Infarction: A Costa Rican Population

**DOI:** 10.1371/journal.pone.0013081

**Published:** 2010-09-29

**Authors:** Lance A. Bare, Edward A. Ruiz-Narvaéz, Carmen H. Tong, Andre R. Arellano, Charles M. Rowland, Joseph J. Catanese, Frank M. Sacks, James J. Devlin, Hannia Campos

**Affiliations:** 1 Celera, Alameda, California, United States of America; 2 Slone Epidemiology Center, Boston University, Boston, Massachusetts, United States of America; 3 Brigham and Women's Hospital, Harvard Medical School, Boston, Massachusetts, United States of America; 4 Department of Nutrition, Harvard School of Public Health, Boston, Massachusetts, United States of America; University of Utah, United States of America

## Abstract

**Background and Methodology:**

The 719Arg allele of *KIF6* (rs20455) was associated with coronary events in Caucasian participants of five prospective studies. We investigated whether this *KIF6* variant was associated with non-fatal myocardial infarction (MI) in a case-control study of an admixed population from the Central Valley of Costa Rica. Genotypes of the *KIF6* variant were determined for 4,134 men and women. Cases (1,987) had survived a first MI; controls (2,147) had no history of MI and were matched to cases by age, sex, and area of residence. We tested the association between the *KIF6* 719Arg allele and non-fatal MI by conditional logistic regression and adjusted for admixture of founder populations.

**Principal Findings:**

Compared with the reference Trp/Trp homozygotes, *KIF6* 719Arg carriers were not at significantly higher risk for non-fatal MI in this study after adjustment for traditional risk factors or admixture (OR = 1.12; 95%CI, 0.98–1.28). Heterozygotes of the *KIF6* Trp719Arg variant were at increased risk of non-fatal MI: the adjusted odds ratio was 1.16 (95% confidence interval, 1.01–1.34), but this association would not be significant after a multiple testing correction.

**Conclusions/Significance:**

We found that carriers of the *KIF6* 719Arg allele were not at increased risk of non-fatal MI in a case-control study of Costa Ricans living in the Central Valley of Costa Rica.

## Introduction

Kinesins are a superfamily of homodimeric motor proteins that transport cellular cargos (e.g., proteins, vesicles, or organelles) along microtubules in an ATP dependent process[1]. A single nucleotide polymorphism (SNP) in the gene for the KIF6 protein—a member of the kinesin 9 family—has been reported to be associated with coronary heart disease (CHD) and event reduction during statin therapy[2]. In Caucasians, carriers of the 719Arg allele of this SNP (rs20455) were at increased risk for coronary events in 5 prospective studies: the Atherosclerosis Risk in Communities (ARIC) study[3], the Cardiovascular Health Study (CHS) [4], the Women's Health Study (WHS)[5], and in the placebo groups of both the Cholesterol and Recurrent Events (CARE) study and the West of Scotland Coronary Prevention Study (WOSCOPS)[2], In contrast to these prospective studies, *KIF6* Trp719Arg was not associated with coronary artery disease (CAD) in two recently reported case-control studies: the Ottawa Heart Genomics study[6] and the Welcome Trust Case-Control Consortium study[7].

Only limited data are available regarding whether the *KIF6* 719Arg allele is associated with CHD in other ethnic populations. For example, among African Americans in ARIC, each allele of *KIF6* 719Arg increased the risk for incident CHD by 1.23-fold (95%CI, 0.99–1.52)[3], and among African Americans in CHS, carriers of the *KIF6* 719Arg allele had a hazard ratio for incident myocardial infarction (MI) of 4.14 (90%CI, 0.79–21.77), compared with noncarriers[4]. Thus, although the associations between the *KIF6* 719Arg allele and CHD were not statistically significant in the small African American study populations in ARIC and CHS, these results suggested that the *KIF6* 719Arg allele may be associated with CHD in ethnic populations other than Caucasians. Therefore, we asked whether the *KIF6* 719Arg allele is associated with MI in a Hispanic population from Costa Rica.

## Results

The characteristics of cases with myocardial infarction and population-based controls from the Central Valley of Costa Rica used in this study are shown in Table 1. Age, sex, and residence (*i.e.*, matched characteristics) did not differ significantly between cases and controls (Table 1). Other CHD risk factors (smoking, history of hypertension, family history of MI, and waist-to-hip ratio) were higher in cases than in controls (Table 1). The genotypes of the *KIF6* SNP (rs20455) that cause a Trp719Arg variation did not deviate from the distribution expected under Hardy–Weinberg equilibrium (*P* = 0.38); the frequency of the minor allele (*KIF6* 719Arg) was 0.36 in the control subjects.

**Table 1:** Characteristics of Cases and Controls.

	Controls	Cases	P Value^1^
Characteristic	(n = 2147)	(n = 1987)	
Age, yrs	58.3±11.3	58.3±11.0	N/A^2^
Male, n (%)	1577 (73)	1474 (74)	N/A^2^
Current Smoker, n (%)	461 (22)	797 (40)	<0.001
Residence (% rural)	546 (25)	517 (26)	N/A^2^
History of diabetes, n (%)	309 (14)	488 (25)	<0.001
History of hypertension, n (%)	634 (30)	771 (39)	<0.001
Family history of MI, n (%)	160 (8)	224 (12)	<0.001
Waist-to-hip ratio	0.95±0.08	0.97±0.07	<0.001

Data are presented as mean ± SD or number (percentages).

1 Difference between cases and controls were tested with the t test for continuous variables and Fisher's exact test for discrete variables.

2 Not applicable because case and controls were matched for this variable.

We found that carriers of one or two copies of the *KIF6* 719Arg variant were not at increased risk of MI (OR = 1.12; 95%CI, 0.98–1.28; Table 2). Although the risk estimate for heterozygotes of the *KIF6* variant (Trp/Arg), compared with major allele homozygotes (Trp/Trp), was 1.15 (95%CI 1.00–1.33; Table 2) after adjusting for potentially confounding risk factors and admixture in this Costa Rican population, this association would not be significant if corrected for the testing of multiple *KIF6* 719Arg carrier groups. Homozygotes of the *KIF6* variant (Arg/Arg) were not at increased risk of MI, compared with major allele homozygotes (Trp/Trp).

**Table 2:** Association of *KIF6* Trp719Arg with Myocardial Infarction.

			Model 1†			Model 2§			Model 3‡		
Genotype	Case	Control	OR	95% CI	p Value	OR	95% CI	p Value	OR	95% CI	p Value
ArgArg + ArgTrp	1202	1251	1.10	0.97–1.25	0.14	1.13	0.98–1.29	0.083	1.12	0.98–1.28	0.10
TrpTrp	785	896	ref			ref			ref		
ArgTrp	952	966	1.13	0.99–1.29	0.074	1.16	1.01–1.34	0.040	1.15	1.00–1.33	0.049
ArgArg	250	285	1.00	0.83–1.22	0.98	1.01	0.82–1.25	0.93	1.00	0.81–1.24	1.00

† Cases and controls were matched for sex, age (±5 y), and county of residence.

§ Adjusted for smoking (current vs. never smoked), history of hypertension, history of diabetes, family history of MI, and waist-to-hip ratio.

‡ Adjusted for Model 2 covariates plus admixture.

OR  =  odds ratio; CI  =  confidence interval; ref  =  reference group.

## Discussion

In contrast to the results reported from 5 prospective studies, we found that *KIF6* 719Arg carriers were not at significantly higher risk for non-fatal MI in this case-control study of a Hispanic population from Costa Rica after adjustment for traditional risk factors or admixture. Heterozygotes of the *KIF6* Trp719Arg variant, compared with Trp/Trp homozygotes, were at increased risk of nonfatal MI (OR = 1.16), but this association would not be significant after a multiple testing correction. Arg/Arg homozygotes of the *KIF6* variant were not at increased risk of MI, compared with Trp/Trp homozygotes.

Although the *KIF6* variant has been found to be associated with CHD in five prospective studies of CHD[2], [3], [4], [5], it was not associated with CAD in two case-control studies: the Ottawa Heart Genomics study[6] and the Wellcome Trust Case-Control Consortium[7]. The Ottawa Heart Genomics case-control study investigated the association between *KIF6* 719Arg and MI in a population with angiographically defined coronary artery disease. The Wellcome Trust Case-Control Consortium analyzed the association between *KIF6* Trp719Arg and CAD. That *KIF6* 719Arg was associated with CHD in prospective, but not in case-control studies, could be explained by either ascertainment biases or by statin use in the populations from which the cases were drawn. Statin use would be expected to reduce the likelihood of observing an association between *KIF6* 719Arg and CHD because in 3 randomized studies[2], [5], statin therapy significantly reduced coronary events in *KIF6* 719Arg carriers but not in noncarriers. The potential inclusion of individuals on statin therapy is also a limitation of our Costa Rica study, which may have weakened the association of the *KIF6* variant with MI.

In previous published prospective studies, risk estimates have been nominally higher for heterozygotes (Trp/Arg) than for homozygotes (Arg/Arg) in most studies of Trp719Arg (WHS, CARE, CHS, and WOSCOPS)[2], [4], [5]. In this study of Costa Ricans, the risk estimates for heterozygotes and homozygotes of *KIF6* 719Arg are consistent with the pattern of these previous studies. The only prospective study where the observed risk estimates have been higher for Arg/Arg homozygotes than heterozygotes of *KIF6* Trp719Arg was the ARIC study[3]. The higher risk estimates for heterozygotes (Trp/Arg) in most studies could be due to chance. Alternatively, the higher risk estimates for heterozygotes could indicate that there is a functional difference between heterodimers and homodimers of the KIF6 protein, possibly because the Arg-Trp heterodimers differ from Arg-Arg and Trp-Trp homodimers in their stability or in their ability to transport cargo. However, the function of the KIF6 protein and its role in CHD remains to be elucidated.

Admixture in the Costa Rican population did not affect the association between *KIF6* 719Arg and MI. Based on historical records[8], [9] and ancestry informative markers[10], [11], [12], the population of the Central Valley of Costa Rica is an admixture of 3 ancestral populations: Southern Europeans, Amerindians and West Africans. The *KIF6* (719Arg) allele frequency of the *KIF6* Trp719Arg variant reported in the HapMap database differs between these populations. The allele frequencies are 38 percent for Caucasians of Western European decent and 95 percent for West Africans (Yoruba residing in Ibadan, Nigeria); the allele frequency for Amerindians from the central valley of Costa Rica was not available from HapMap. Since the *KIF6* 719Arg allele frequency varies according to ancestry, a spurious association could occur if the admixture of the 3 ancestral populations were different between cases and controls. However, adjustment for the degree of admixture did not appreciably change the *KIF6* 719Arg risk estimate for MI.

### Limitations

Since participants with cardiovascular diseases other than a history of MI were not excluded from the control group, the risk estimates obtained in this study may be lower than would have been observed had a disease-free control group been used. The use of statins was previously reported to ameliorate the risk of coronary events in carriers of *KIF6* 719Arg in CARE and WOSCOPS[2]. Therefore, statin use in the population might cause the risk estimates for MI in this study to be underestimated; however, information on statin use among this population was not available.

### Conclusions

Carriers of the *KIF6* 719Arg allele were not at increased risk of non-fatal MI, compared with Trp/Trp homozygotes, in a case-control study from the Central Valley of Costa Rica. We found no evidence that admixture within the Costa Rican population affected these results. Additional studies are need to address why the association between carriers of the *KIF6* 719Arg and MI differ between this and other case-control studies and the previously reported prospective studies.

## Methods

### Study Population

The design of the original population-based case-control study has been described in detail elsewhere[13]. The catchment area for this study comprised 7071 km^2^ and 2,057,000 culturally Hispanic individuals living in Costa Rica. This area included 36 counties in the Central Valley of Costa Rica representing a full range of socioeconomic levels, as well as urban, periurban, and rural lifestyles. Medical services for all residents of this area were provided by 6 large hospitals, which are part of the National Social Security System. Eligible case participants were men and women who were survivors of a first MI as diagnosed by a cardiologist at any of the 6 recruiting hospitals in the catchment area between 1994 and 2004. Since this study was designed to draw cases and controls who were representative of the general population and the intent was that cases should differ from controls only by the case definition, the presence of other cardiovascular conditions was not used to exclude controls (or cases). Control participants were randomly selected using data from the National Census and Statistics Bureau of Costa Rica. Control participants were ineligible if they had ever had an MI. The controls in this study were matched to cases by assigning participants to strata based on age groups (±5 y), sex, and area of residence (county). Four controls were excluded from the study because they were assigned to strata for which there were no matching cases, resulting in 1987 cases and 2147 controls. Study and consent documents were approved by the Human Subjects Committee of both the Harvard School of Public Health and the University of Costa Rica—all participants gave written informed consent.

### Genotype Determination

*KIF6* Trp719Arg genotypes were determined for all study participants using an allele–specific real-time PCR genotyping assay[14] at a core facility. The DNA was standardized to 10 ng/µL using PicoGreen (Molecular Probes, Invitrogen Corp, Carlsbad, CA) fluorescent dye. The Trp719Arg genotypes determined with this assay have been shown to be >99.8% concordant with genotypes determined using an alternative genotyping technology[4]. The primer sequences are available upon request. Genotypes for the 39 ancestry informative markers (AIMs) listed in the online supplement, Table S1, were previously ascertained[12].

The admixture in this study population was previously determined using the program ADMIXMAP and 39 AIMs to estimate the proportion of admixture of the 3 ancestral ethnicities for each subject[12]. The 39 AIMs were selected because they showed large allelic frequency differences among the three founding ancestral populations of the Costa Rican Central Valley: Southern European, West-African and Amerindian[10], [15], [16], [17].

### Statistical Analysis

Differences between baseline characteristics of patients were assessed by the t-test (for continuous variables) or by the χ^2^ test (for discrete variables). Deviations from Hardy–Weinberg expectations were assessed using an exact test in controls. Conditional logistic regression models were used to assess the association between genotype and risk of MI while taking into account the matching for age group, sex, and area of residence. SAS version 9 software was used for all regression models. Models were adjusted for smoking status (current vs. past or never smoked)[18], history of hypertension (yes or no), history of diabetes (yes or no), waist-to-hip ratio (quintiles based on the distribution in controls), family history of MI (at least one of the parents had an MI before the age of 60) and admixture (proportion of Amerindian and West-African ancestral ethnicities). We did not adjust for plasma lipids because pre-MI plasma lipid levels were not available. The available plasma lipid levels were determined after the MI event and may have been affected by the MI event, therapy or changes in lifestyle. All *P*-values are 2-sided.

## Acknowledgments

We are grateful to the study participants, to Xinia Siles for data collection and study management in Costa Rica, and to the staff of Proyecto Salud Coronaria, San José, Costa Rica. We also thank the investigators who participated in the collection of the clinical samples and Nam Bui, David Ross, and David Wolfson for expert technical support.

## Supporting Information

Table S1 39 Ancestry Informative Markers
(0.09 MB DOC)
