# Supplementary material for: Investigation of KIF6 Trp719Arg in a Case-Control Study of Myocardial Infarction: A Costa Rican Population
Source: PLoS One. 2010 Sep 29;5(9):e13081. doi: 10.1371/journal.pone.0013081 (PMC2947524; doi:10.1371/journal.pone.0013081)
Supplement: Table S1 — 39 Ancestry Informative Markers (0.09 MB DOC) [file pone.0013081.s001.doc]

**Table S1**. 39 Ancestry Informative Markers

| **dbSNP id** | **Chr** | **Position** | **Gene symbol** |
| --- | --- | --- | --- |
| rs1240709 | 1 | 1327700 | MRPL20 |
| rs2156087 | 1 | 23616168 | TCEA3 |
| rs140864 | 1 | 36164250 | EIF2C1 |
| rs2814778 | 1 | 157441307 | DARC |
| rs2065160 | 1 | 203057600 |  |
| rs723632 | 1 | 212126375 |  |
| rs880143 | 1 | 245934736 |  |
| rs9309717 | 2 | 3474085 | TTC15 |
| rs3768641 | 2 | 72221698 | CYP26B1 |
| rs4596126 | 3 | 13634898 | FBLN2 |
| rs17203 | 3 | 76407961 |  |
| rs758973 | 4 | 13148471 |  |
| rs7041 | 4 | 72837198 | GC |
| rs16344 | 4 | 101620192 | EMCN |
| rs3309 | 5 | 56128536 |  |
| rs3317 | 5 | 112240050 | APC |
| rs2227282 | 5 | 132041078 | IL4 |
| rs3340 | 5 | 153812060 | SAP30L |
| rs2763 | 7 | 556186 | LOC729623 |
| rs2965404 | 7 | 21716027 |  |
| rs1985080 | 7 | 33593384 | BBS9 |
| rs2161 | 7 | 97930442 |  |
| rs1871534 | 8 | 145610489 | SLC39A4 |
| rs2695 | 9 | 82074397 |  |
| rs992528 | 10 | 118152572 |  |
| rs905552 | 11 | 19052735 |  |
| rs1042602 | 11 | 88551344 | TYR |
| rs1800498 | 11 | 112796798 | DRD2 |
| rs1079598 | 11 | 112801484 | DRD2 |
| rs8009244 | 14 | 54713505 | DLG7 |
| rs2714758 | 15 | 23030430 |  |
| rs1800404 | 15 | 25909368 | OCA2 |
| rs2862 | 15 | 32932845 |  |
| rs1426654 | 15 | 46213776 | SLC24A5 |
| rs4646 | 15 | 49290136 | CYP19A1 |
| rs2228478 | 16 | 88514109 | MC1R |
| rs4884 | 19 | 50501875 | CKM |
| rs722098 | 21 | 15607469 |  |
| rs183564 | 21 | 16561693 | C21orf34 |

SNPs were selected from the literature[1,2,3]

**References**

1. Shriver MD, Parra EJ, Dios S, Bonilla C, Norton H, et al. (2003) Skin pigmentation, biogeographical ancestry and admixture mapping. Hum Genet 112: 387-399.

2. Smith MW, Patterson N, Lautenberger JA, Truelove AL, McDonald GJ, et al. (2004) A high-density admixture map for disease gene discovery in African Americans. Am J Hum Genet 74: 1001-1013.

3. Bonilla C, Parra EJ, Pfaff CL, Dios S, Marshall JA, et al. (2004) Admixture in the Hispanics of the San Luis Valley, Colorado, and its implications for complex trait gene mapping. Ann Hum Genet 68: 139-153.
